# Oral health literacy and socio-demographics as determinants of oral health status and preventive behavior measures in participants of a pre-marriage counseling program

**DOI:** 10.1371/journal.pone.0258810

**Published:** 2021-11-05

**Authors:** Maryam Fazli, Reza Yazdani, Simin Zahra Mohebbi, Ahmad Reza Shamshiri

**Affiliations:** 1 Research Center for Caries Prevention (RCCP), Dental Research Institute, Tehran University of Medical Sciences, Tehran, Iran; 2 Department of Community Oral Health, School of Dentistry, Tehran University of Medical Sciences, Tehran, Iran; Anglia Ruskin University, UNITED KINGDOM

## Abstract

This analytical cross-sectional study aimed to assess the correlation of oral health literacy (OHL) and oral health status and behavior in participants of a pre-marriage counseling program. Also, it aimed to determine the target population’s OHL needs to design necessary interventions. A total of 828 couples with a mean age of 24.22 years participating in a pre-marriage counseling program were evaluated. Data were collected using the oral health literacy-adult questionnaire (OHL-AQ). Clinical oral examinations were performed to measure the gingival index (GI), plaque index (PI), and DMFT. The mean OHL score of the couples was 7.86±3.83 out of 17; while, the mean DMFT score was 6.30±5.73. After controlling for the confounders, OHL had a significant correlation with the frequency of dental visits, and smoking status, and inverse correlation with the number of decayed teeth and missing teeth, and PI, and GI (p<0.05). The current results highlight the necessity of OHL interventions to fill the existing gaps. This is an unmet need of the couples, and such interventions are required to promote their own oral health as well as the oral health of their future children.

## Introduction

In the recent years, knowledge about the oral health literacy (OHL) for promotion of oral health has increased. OHL is a key element in strategy planning for oral health promotion [1, 2]. Attempts are ongoing to integrate the concept of OHL into practice and oral health research [1]. Healthy People 2010 defines the OHL as the “degree to which individuals have the capacity to obtain, process, and understand basic oral health information and services needed to make appropriate health decisions”. OHL can serve as a link between the socioeconomic factors and behavioral and health outcomes [1, 3]. Patients with poor OHL have higher frequency of carious teeth and extracted teeth, and higher prevalence of periodontal disease [4, 5]. Also, evidence shows considerably high prevalence of inadequate OHL in adults [6–8].

On the other hand, family plays an important role in general health of a community, and health promotion should start from the family level. After marriage, the couples share the same environmental and social characteristics. It has been reported that living together causes couples to have the same behaviors (behavioral convergence) [9]. Health interventions mostly target females because they are more easily accessible as the target population [10]. This may lead to an imbalance in OHL of the young couples. To overcome this, OHL interventions should equally target males and females. Couples are an ideal target group for such interventions because they can transmit diseases to each other or to their future children. Transmission of human papilloma virus by oral sex is among such diseases [11, 12]. Also, they can affect the oral health behavior of each other and their future children [11].

Interventions should be started early enough to have maximum efficacy. Thus, pre-marriage counseling programs may provide an ideal opportunity for oral health interventions. Since marriage occurs among individuals from all socioeconomic levels, such interventions can target a large part of the community. According to the National Organization for Civil Registration, averagely 750,000 marriages occur annually in Iran [13]. The newlyweds should be familiarized with the preventive measures and the fact that oral conditions are mostly preventable. According to the Ministry of Health in Iran [14], pre-marriage counseling programs are mandatory for all new couples. Thus, such programs can provide a great opportunity for provision of interventions for enhancement of OHL of the couples. However, first, the OHL of the couples should be measured and their oral health-related social, behavioral and epidemiological factors, as well as their requirements as the target population should be assessed.

Since the available OHL interventions for improvement of health outcomes are limited, and the implemented interventions have been mostly performed on small and vulnerable populations [15], we planned a project named “Save Couples’ Smile” with community orientation. Thus, this study, as the primary step of this project, aimed to assess the OHL and socio-demographics as determinants of oral health status and preventive behavior measures in participants of a pre-marriage counseling program. Also, it aimed to determine the target population’s OHL needs to design necessary interventions.

## Materials and methods

This analytical cross-sectional study comprised the first phase of a 4-phase project named “Save Couples’ Smile” that was started in 2018 on young couples participating in pre-marriage counseling programs in Zanjan, the capital city of a province with 1 million population located in the north-west of Iran. According to the Ministry of Health of Iran, pre-marriage counseling programs as part of the primary healthcare system are mandatory for all couples. The study protocol was approved by the Ethics Committee of Tehran University of Medical Sciences (IR.TUMS.DENTISTRY.REC.1397.068). The “Save Couples’ Smile” project is an interventional OHL project designed according to the Precede-Proceed model for public oral health promotion. This study was the social and epidemiological phase of our planned model, the PRECEDE/PROCEED model, which was conducted along with a need assessment to find the existing gaps and design an effective content for future OHL interventions.

The sample size calculation was carried out for a dual purpose; firstly, to perform the current cross-sectional study, and secondly to have adequate sample size to perform the implementation phase of the model as a randomized controlled clinical trial. The sample size was calculated to be 576 individuals considering adequate OHL of 0.4, precision of 0.04, and 95% confidence interval using the sample size calculation formula by Daniel et al [16]. The calculated sample size increased by 30% to achieve the required sample size to execute the next phase of the project. Participants were selected by purposeful convenience sampling. Males and females presenting to the only pre-marriage counseling center in Zanjan city were invited and enrolled in the study to complete the sample size. They were considered as independent units in this study since they had not yet started their life together. The basis of allocation of individuals to the intervention and control groups was based on their day of attendance to the pre-marriage health center. From 902 individuals that were invited to participate in the study, 828 accepted to participate (92%). Up on the last marriage statistics of the province 6900 cases of marriage were occurred, so these numbers of participants would be representative [13].

The inclusion criteria were age between 15–35 years, the ability to speak, read and write in Farsi, and signing informed consent forms. Participation in the study was voluntary. The study was conducted in accordance with the Declaration of Helsinki. Participants under the age of 16 were considered as minors. Thus, their parents gave written informed consent for their participation in the study. The study objectives were thoroughly explained to the participants both verbally and written, and also by putting on a billboard at the study location. They were also informed that they could quit whenever they wished to do so.

Data regarding the OHL, oral health behaviors, and demographic variables were collected by using an OHL questionnaire. We used the oral health literacy-adult questionnaire (OHL-AQ), which is a valid and reliable questionnaire for this purpose [17]. The questionnaire was filled out by a combination of self-report by the participants and an interview by a trained family health technician. The questionnaire had 17 items for assessment of OHL in four domains of health literacy skills: print literacy (reading and writing), numeracy skills, oral literacy skills (listening), and information seeking (navigation of Internet and facilities) toward health decision making [18]. The OHL was categorized into three levels of inadequate (scores 0–9), marginal (scores 10–11), and adequate (scores 12–17) [6, 15]. Oral health behaviors were evaluated in four domains of tooth brushing, dental check-up, use of sugary snacks, and smoking status. Oral health behaviors were classified based on evidence [19, 20]. Regarding the sugary snack consumption, there were two answer choices of "less than twice a day" and "twice a day or more”. For the frequency of toothbrushing, there were two answer choices of “twice a day or more” and “less than twice a day”. Age (categorized as > 22 and ≤ 22 years [21]), gender, place of residence, floor area per person (as an index of economic status) [22], and years of education (as an index of social class) were recorded as the demographic characteristics of the participants.

Clinical oral examinations included measurement of DMFT, plaque index (PI), and gingival index (GI). Examinations were performed by a dentist using a dental mirror, a dental explorer, and a periodontal probe on a dental chair. Twenty-five participants were evaluated again by the same dentist to assess the intra-examiner reliability (kappa = 0.93). The DMFT of the participants was determined according to the WHO criteria for oral health studies [23]. The PI and GI were measured according to the Loe and Silness criteria [24].

Descriptive statistics were applied, and the mean and percentage values were reported for demographic variables, dental and gingival health indices (DMFT, DT, MT, FT, PI and GI), oral health behaviors, and OHL. Considering the distribution of data, generalized linear models were used for quantitative data, and logistic and ordered regression for qualitative data to assess the correlation of dependent variables with OHL after controlling for the demographic factors. The backward method in multivariate analysis was applied to control for the effect of confounders and their interactions. The normality of data distribution was analyzed using the Kolmogorov-Smirnov test and the Shapiro-Wilk test. Level of significance was set at 0.05. All statistical analyses were carried out using STATA version 14 software (STATA Corp., 2015; TX, USA).

## Results

A total of 828 participants were enrolled. The response rate to the OHL-AQ was 100%, and all participants underwent clinical oral examination. The mean age of the participants was 24.22±5.44 years (range 15 to 35 years). The level of education was lower than high-school diploma (< 12 years of education) in 36.2% of the participants. The mean household size was 4.29±1.32 members. Of participants, 49.6% reported living in houses smaller than 20 square-meters per each individual. The mean score of OHL was 7.86±3.83 out of 17, with a median of 8. Of all participants, 64% had inadequate OHL, 15% had marginal OHL, and 21% had adequate OHL. Also, 56.4% of those ≥ 22 years of age had adequate OHL. Of those with adequate OHL, 81.8% had an educational level equal or higher than high-school diploma (≥ 12 years of education); 51.4% were living in urban areas. The findings also showed that participants with inadequate OHL had higher frequency of dental visits due to toothache as well as emergency dental treatments.

Clinical oral examination revealed a mean DMFT score of 6.30±5.73. The mean number of decayed teeth was 2.19±3.39, the mean number of missing teeth was 1.52±3.76, and the mean number of filled teeth was 2.59±3.36. The mean PI was 1.36±0.61, and the mean GI was 1.29±0.66 out of 3. Also, 89% of the participants were non-smokers, 30.7% reported toothbrushing twice a day, and 33.3% reported having sugary snacks more than once a day. According to the results, the OHL and demographic factors were correlated with oral health preventive behaviors and oral health status in some cases. Certain demographic factors were correlated with oral health preventive behaviors and oral health status. No such correlations were found between age and tooth brushing, dental check-ups, and smoking, or between gender and dental check-ups. Education, while not correlated with DMFT, showed a significant relationship with its components, namely DT, MT, and FT. Also, gender showed a strong significant correlation with PI and GI. However, no such a correlation was found between the mean number of filled teeth, frequency of taking sugary snacks, or frequency of daily toothbrushing with OHL. We examined the interaction effects by ANOVA/post-hoc, which revealed no significant interaction effect (p>0.05); thus, the interpretation of the main effects was correct and not misleading.

Table 1 shows the oral health preventive behavior of the participants based on their demographics and OHL, and the relationship of OHL and oral health behaviors according to the univariate analysis and also after controlling for other variables and using the backward multivariate analysis. As shown, after adjusting for the confounders, adequate OHL was found to be associated with the participants’ smoking behavior. While, utilization of dental services was correlated with all three levels of OHL.

**Table 1:** Oral health literacy and demographic characteristics as well as their relationship with oral health preventive behaviors of the participants (n:828).

		Descriptive data			Unadjusted univariate analysis			Adjusted multivariate analysis, backward method		
		N (%)			OR(SE)	95%CI	p-value	OR(SE)	95%CI	p-value
**Dental brushing** ^1^		Never	≤Once daily	≥Twice daily						
**Gender**	Male	89(10.8)	247(30.0)	71(8.6)	Ref.			Ref.		
	Female	162(19.7)	232(28.2)	23(2.8)	2.80(0.47)	2.02, 3.84	**< 0.001**	2.89(0.43)	2.17, 3.86	**< 0.001**
**Age (years)**	< 22	105(12.7)	181(22.0)	27(3.3)	Ref.					
	≥ 22	146(17.7)	298(36.2)	67(8.1)	0.94(0.16)	0.66, 1.32	0.71			
**Years of education**	< 12	152(18.5)	302(36.6)	82(9.9)	Ref.			Ref.		
	≥ 12	99(12.0)	177(21.5)	12(1.5)	1.37(0.24)	0.97, 1.93	**0.07**	1.44(0.23)	1.06, 1.98	**0.020**
**Floor area/person**	< 20	112(13.6)	249(29.9)	49(6.0)	1.22(0.12)	1.00, 1.48	**0.05**			
	20–39	85(10.3)	158(19.2)	35(4.2)				1.24(0.12)	1.01, 1.50	**0.03**
	≥ 40	54(6.5)	75(9.1)	10(1.2)						
**Place of residence**	Urban	168(20.4)	303(36.8)	42(5.1)	Ref.			Ref.		
	Suburban	39(4.7)	70(8.5)	15(1.8)	1.10(0.23)	0.73, 1.64	0.65	1.06(0.22)	0.71, 1.68	0.768
	Rural	44(5.3)	106(12.9)	37(4.5)	0.60(0.11)	0.42, 0.87	**0.007**	0.58(0.11)	0.40, 0.83	**0.003**
**Oral health literacy**	Inadequate	148(18.0)	301(36.5)	76(9.2)	Ref.					
	Marginal	39(4.7)	78(9.5)	10(1.2)	1.13(0.23)	0.76, 1.69	0.61			
	Adequate	64(7.8)	100(12.1)	8(1.0)	1.27(0.25)	0.88, 1.88	1.21			
**Dental check-up** ^2^		Within the past 6 months		More than 6 months ago						
**Gender**	Male	133(16.1)		277(33.5)	Ref.					
	Female	136(16.4)		282(34.1)	1.04(0.18)	0.74, 1.47	0.81			
**Age (years)**	< 22	98(11.8)		217(26.2)	Ref.					
	≥ 22	171(26.7)		342(41.3)	1.17(0.23)	0.80, 1.71	0.41			
**Years of education**	< 12	147(17.8)		393(47.4)	Ref.			Ref.		
	≥ 12	122(14.7)		166(20.1)	0.65(0.12)	0.45, 0.94	**0.02**	0.67(0.12)	-0.47, -0.94	**0.022**
**Floor area/person**	< 20	114(13.8)		295(35.6)						
	20–39	107(12.9)		173(20.9)	0.93(0.10)	0.75, 1.15	0.50			
	≥ 40	48(5.8)		91(11.0)						
**Place of residence**	Urban	190(23.0)		325(39.2)	Ref.					
	Suburban	30(3.6)		95(11.5)	1.55(0.36)	0.97, 2.45	0.06			
	Rural	49(5.9)		139(16.8)	1.22(0.25)	0.81, 1.82	0.35			
**Oral health literacy**	Inadequate	142(17.1)		386(46.6)	Ref.			Ref.		
	Marginal	52(6.3)		75(9.1)	0.64(0.14)	0.42, 0.97	**0.036**	0.63(0.13)	0.41,0.96	**0.033**
	Adequate	75(9.1)		98(11.8)	0.65(0.19)	0.43, 0.98	**0.041**	0.64(0.13)	0.43,0.96	**0.033**
**Sugary snack consumption** ^2^		≥Twice/day		<Twice/day						
**Gender**	Male	220(26.6)		190(23.0)	Ref.			Ref.		
	Female	216(26.1)		202(24.3)	1.40(0.23)	1.01, 1.93	**0.04**	1.39(0.23)	1.00,1.91	**0.047**
**Age (years)**	< 22	178(21.5)		137(16.5)	Ref.			Ref.		
	≥ 22	258(31.2)		255(30.8)	1.71(0.31)	1.20, 2.43	**0.003**	1.73(0.31)	1.22,2.45	**0.002**
**Years of education**	< 12	272(32.9)		268(32.3)	Ref.			Ref.		
	≥ 12	164(19.8)		124(15.0)	0.65(0.12)	0.46, 0.93	**0.02**	0.66(0.10)	-0.48,-0.89	**0.007**
**Floor area/person**	< 20	216(26.1)		193(23.3)						
	20–39	143(17.3)		137(16.5)	1.00(0.10)	0.82, 1.22	0.99			
	≥ 40	77(9.3)		62(7.5)						
**Place of residence**	Urban	271(32.7)		244(29.5)	Ref.					
	Suburban	62(7.5)		63(7.6)	1.03(0.21)	0.69, 1.54	0.90			
	Rural	103(12.4)		85(10.3)	0.82(0.15)	0.57, 1.19	0.30			
**Oral health literacy**	Inadequate	271(32.7)		257(31.1)	Ref.					
	Marginal	69(8.3)		58(7.0)	0.95(0.20)	0.63, 1.42	0.80			
	Adequate	96(11.6)		77(9.3)	0.90(0.18)	0.60, 1.36	0.59			
**Smoking** ^2^		No		Yes						
**Gender**	Male	338(40.8)		72(8.70)	Ref.			Ref.		
	Female	403(48.7)		15(1.80)	0.18(0.06)	0.09, 0.35	**<0.001**	0.17(0.50)	0.096,0.30	**<0.001**
**Age (years)**	< 22	296(35.8)		19(2.3)	Ref.					
	≥ 22	445(53.7)		68(8.2)	1.10(0.35)	0.58, 2.06	0.77			
**Years of education**	< 12	481(58.1)		59(7.1)	Ref.					
	≥ 12	260(31.4)		28(3.4)	1.13(0.33)	0.63, 2.01	0.68			
**Floor area/person**	< 20	364(44.0)		45(5.4)						
	20–39	257(31.0)		23(2.8)	0.98(0.16)	0.71, 1.36	0.91			
	≥ 40	120(14.5)		19(2.3)						
**Place of residence**	Urban	465(56.2)		50(6.0)	Ref.					
	Suburban	105(12.7)		20(2.4)	1.36(0.42)	0.74, 2.48	0.32			
	Rural	171(20.6)		17(2.1)	0.83(0.26)	0.44, 1.55	0.55			
**Oral health literacy**	Inadequate	462(55.8)		66(8.0)	Ref.			Ref.		
	Marginal	117(14.1)		10(1.2)	0.52(0.20)	0.25, 1.09	**0.08**	0.55(0.2)	0.27,1.11	0.099
	Adequate	162(19.6)		11(1.3)	0.43(0.16)	0.20, 0.91	**0.03**	0.46(0.16)	0.23,0.90	**0.025**

^1^Ordered logistic regression

^2^Binary logistic regression

OR: odds ratio, CI: Confidence interval, SE: Standard error

It should be noted that the frequency of toothbrushing in females was 2.89 times more than that in males. Also, the frequency of toothbrushing was 44% higher in those with high-school diploma and university education compared with those with an educational level lower than high-school diploma. Frequency of toothbrushing was higher by 24% in those with smaller living area, and by 58% in rural inhabitants (compared with urban inhabitants). Frequent use of dental services (≤6 months) was 36% higher in those with adequate OHL. Also, the frequency of non-smokers was 54% higher in those with adequate OHL.

Table 2 shows the dental health status of the participants based on their demographics and OHL score, and the correlation of OHL and dental health status according to the univariate and multivariate analysis after controlling for other variables using the backward method. As shown in Table 2, there were significant associations between the OHL level and clinical dental parameters, except for the FT (filled teeth) after adjusting for possible confounding factors.

**Table 2:** Oral health literacy and demographic characteristics and their relationships with dental health status of the study participants in SCS (Save Couples’ Smile) study (n:828).

		Descriptive data Mean(SD)	Unadjusted univariate analysis			Adjusted multivariate analysis, backward method		
			Coef.(SE)^1^	95%CI	p-value	Coef.(SE)^1^	95%CI	p-value
**DMFT**								
**Gender**	Male	7.54(6.44)	Ref.			Ref.		
	Female	5.08(4.62)	-0.15(0.07)	-0.29, -0.02	**0.030**	-0.15(0.07)	-0.29, -0.01	**0.032**
**Age (year)**	< 22	3.91(3.66)	Ref.			Ref.		
	≥ 22	7.77(6.25)	0.60(0.07)	0.45, 0.75	**<0.001**	0.59(0.07)	0.45, 0.74	**<0.001**
**Years of education**	< 12	5.94(6.14)	Ref.					
	≥ 12	6.96(4.81)	0.01(0.08)	-0.14, 0.16	0.866			
**Residential surface area/person**	< 20	6.09(5.99)						
	20–39	6.20(5.48)	0.93(0.10)	0.75, 1.15	0.50			
	≥ 40	7.13(5.40)						
**Residency**	Urban	6.70(5.72)	Ref.			Ref.		
	Suburban	6.32(5.76)	-0.11(0.09)	-0.28, 0.07	0.237	-0.10(0.09) -	-0.27,0.08	0.269
	Rural	5.19(5.60)	-0,20(0.08)	-0.37, -0.04	**0.013**	0.18(0.08)	-0.33,-0.02	**0.023**
**Oral health literacy**	Inadequate	6.26(6.28)	Ref.					
	Marginal	5.89(4.26)	-0.12(0.09)	-0.31, 0.06	0.184			
	Adequate	6.72(4.84)	-0.04(0.09)	-0.21, 0.14	0.691			
**Decayed Teeth**								
**Gender**	Male	2.64(3.98)	Ref.			Ref.		
	Female	1.75(2.63)	-0.26(0.11)	-0.47,-0.04	**0.017**	-0.26(0.11)	-0.47,-0.05	**0.016**
**Age (year)**	< 22	1.60(2.10)	Ref.			Ref.		
	≥ 22	2.56(3.94)	0.46(0.12)	0.22, 0.69	**<0.001**	0.45(0.12)	0.23, 0.68	**<0.001**
**Years of education**	< 12	2.38(3.64)	Ref.			Ref.		
	≥ 12	1.84(2.85)	-0.29(0.12)	-0.52,-0.06	**0.015**	-0.29(0.12)	-0.52, -0.05	**0.014**
**Residential surface area/person**	< 20	2.46(3.64)	-0.13(0.07)	-0.26,-0.004	**0.042**			
	20–39	1.80(2.93)				-0.13(0.07)	0.26,-0.005	**0.041**
	≥ 40	2.20(3.49)						
**Residency**	Urban	2.14(3.57)	Ref.					
	Suburban	2.28(2.95)	0.04(0.14)	-0.26, 0.27	0.978			
	Rural	2.28(3.18)	0.006(0.12)	-0.23, 0.25	0.955			
**Oral health literacy**	Inadequate	2.41(3.68)	Ref.			Ref.		
	Marginal	1.51(1.97)	-0.38(0.14)	-0.66,-0.09	**0.008**	-0.38(0.14)	-0.66,-0.10	**0.008**
	Adequate	2.03(3.25)	-0.02(0.13)	-0.28,0.25	0.894	-0.02(0.13)	-0.28,0.24	0.887
**Missing Teeth**								
**Gender**	Male	2.12(4.74)	Ref.			Ref.		
	Female	0.90(2.30)	-0.29(0.14)	-0.57, -0.009	**0.013**	-0.28(0.14)	-0.56, -0.009	0.052
**Age (year)**	< 22	0.53(1.07)	Ref.			Ref.		
	≥ 22	2.11(4.60)	1.36(0.16)	1.04,1.67	**<0.001**	1.35(0.16)	1.04,1.67	**<0.001**
**Years of education**	< 12	1.79(4.45)	Ref.			Ref.		
	≥ 12	0.98(1.78)	-0.56(0.16)	-0.87, -0.24	**0.001**	-0.59(0.16)	-0.90, -0.28	**0.001**
**Residential surface area/person**	< 20	1.67(4.24)						
	20–39	1.40(3.51)	-0.11(0.09)	-0.29, 0.06	0.213			
	≥ 40	1.26(2.54)						
**Residency**	Urban	1.34(3.26)	Ref.					
	Suburban	1.92(4.97)	-0.06(0.18)	-0.28, 0.41	0.735			
	Rural	1.70(4.10)	0.20(0.16)	-0.30, 0.33	0.899			
**Oral health literacy**	Inadequate	1.85(4.54)	Ref.			Ref.		
	Marginal	1.01(1.63)	-0.31(0.20)	-0.69,0.08	0.115	-0.32(0.19)	-0.69,0.06	0.103
	Adequate	0.84(3.25)	-0.44(0.19)	-0.82,-0.07	**0.022**	-0.49(0.19)	-0.86,-0.12	**0.009**
**Filled Teeth**		Mean(SD)						
**Gender**	Male	2.77(3.44)	Ref.					
	Female	2.40(3.29)	-0.03(0.12)	-0.26, 0.20	0.81			
**Age (year)**	< 22	1.77(2.76)	Ref.			Ref.		
	≥ 22	3.09(3.59)	0.24(0.13)	-0.018,0.49	0.07	0.25(0.12)	0.02,0.48	**0.03**
**Years of education**	< 12	1.76(2.87)	Ref.			Ref.		
	≥ 12	4.13(3.67)	0.54(0.12)	0.29,0.78	**<0.001**	0.58(0.12)	0.35,-0.81	**<0.001**
**Residential surface area/person**	< 20	1.15(2.90)						
	20–39	2.99(3.67)	0.13(0.07)	-0.01,0.27	0.08			
	≥ 40	3.66(3.60)						
**Residency**	Urban	3.20(3.66)	Ref.			Ref.		
	Suburban	2.10(2.72)	-0.29(0.15)	-0.60,0.008	0.056	-0.32(0.15)	-0.62,-0.02	**0.036**
	Rural	1.21(2.30)	-0.67(0.14)	-0.95,-0.39	**<0.001**	-0.71(0.14)	-0.98,-0.43	**<0.001**
**Oral health literacy**	Inadequate	1.99(2.99)	Ref.					
	Marginal	3.35(3.45)	0.14(0.15)	-0.16, 0.44	0.365			
	Adequate	3.85(3.90)	0.16(0.14)	-0.12, 0.44	0.261			

Individuals with inadequate OHL were more likely to fall into a higher DT (decayed teeth) and MT (missing teeth) categories compared with those with high OHL. The likelihood of having decayed teeth was 32% lower in those with marginal OHL compared with those with inadequate OHL (P = 0.008). Also, the likelihood of having missing teeth was 61% lower in those with adequate OHL compared with those with inadequate OHL (P = 0.022). By each one unit increase in living space/person, the likelihood of DT decreased by 12% (P = 0.04).

Table 3 shows the oral hygiene and gingival health status of the participants based on their demographics and OHL score, and the correlation of OHL with oral hygiene and gingival health status according to the univariate and multivariate analysis after controlling for other variables using the backward method. As shown in Table 3, there were significant associations between the level of OHL and clinical gingival parameters and oral hygiene after adjusting for the possible confounding variables. Those with lower level of OHL had poorer oral hygiene and more severe gingivitis.

**Table 3:** Oral health literacy, demographic characteristics and their relationship with oral hygiene and gingival health status of the participants (n:828).

		Descriptive data N (%)			Unadjusted univariate analysis			Adjusted multivariate analysis, backward method		
					OR (SE) ^1^	95%CI	p-value	OR (SE) ^1^	95%CI	p-value
**Plaque Index**		Good	Moderate	Fair						
**Gender**	Male	37(4.5)	271(32.9)	99(12.0)	Ref.			Ref.		
	Female	77(9.3)	281(34.0)	60(7.3)	0.50(0.09)	0.35,0.69	**<0.001**	0.45(0.07)	0.33, 0.61	**<0.001**
**Age (years)**	< 22	48(5.8)	215(26.1)	52(6.3)	Ref.					
	≥ 22	66(8.0)	337(40.8)	167(13.0)	1.30(0.24)	0.91,1.87	0.153			
**Years of education**	< 12	59(7.1)	352(42.7)	126(15.3)	Ref.			Ref.		
	≥ 12	55(6.7)	200(24.2)	33(4.00)	0.59(0.11)	0.41,0.86	**0.006**	0.61(0.11)	0.43, 0.87	**0.007**
**Floor area/person**	< 20	40(4.8)	278(33.7)	90(10.9)						
	20–39	48(5.8)	183(22.2)	47(5.7)	0.80(0.09)	0.65,0.99	**0.041**	0.80(0.08)	0.65, 0.98	**0.035**
	≥ 40	26(3.2)	91(11.0)	22(2.7)						
**Place of residence**	Urban	82(9.9)	345(41.8)	88(10.7)	Ref.					
	Suburban	14(1.7)	99(10.8)	20(2.4)	0.83(0.17)	0.55,1.27	0.393			
	Rural	18(2.2)	118(14.3)	51(6.2)	1.35(0.26)	0.92,1.98	0.121			
**Oral health literacy**	Inadequate	63(7.6)	334(40.5)	128(15.5)	Ref.			Ref.		
	Marginal	19(2.3)	89(10.8)	19(2.3)	0.75(0.16)	0.49,1.15	0.190	0.73(0.16)	0.47, 1.11	0.143
	Adequate	32(3.9)	129(15.6)	12(1.5)	0.59(0.12)	0.39,0.89	**0.012**	0.58(0.12)	0.38, 0.87	**0.008**
**Gingival Index**		Mild	Moderate	Severe						
**Gender**	Male	127(15.4)	217(26.3)	63(7.6)	Ref.			Ref.		
	Female	195(23.7)	198(24.0)	25(3.0)	0.51(0.82)	0.37,0.70	**<0.001**	0.51(0.82)	0.37,0.70	**<0.001**
**Age (years)**	< 22	137(16.6)	158(19.2)	20(2.4)	Ref.			Ref.		
	≥ 22	185(22.4)	257(31.2)	68(8.2)	1.44(0.25)	1.02, 2.02	**0.038**	1.43(0.25)	1.02, 2.02	**0.038**
**Years of education**	< 12	192(23.3)	273(33.1)	72(8.7)	Ref.					
	≥ 12	130(15.8)	142(17.2)	16(1.9)	0.73(0.13)	0.52,1.02	0.065			
**Floor area /person**	< 20	142(17.2)	213(25.8)	53(6.4)						
	20–39	117(14.2)	137(16.6)	24(2.9)	0.80(0.79)	0.66,0.97	**0.023**	0.79(0.79)	0.66,0.97	**0.023**
	≥ 40	63(7.7)	65(7.9)	11(1.3)						
**Place of residence**	Urban	222(26.9)	248(20.1)	45(5.5)	Ref.			Ref.		
	Suburban	44(5.3)	71(8.6)	8(1.0)	0.99(0.20)	0.67,1.4	0.954	0.99(0.20)	0.67,1.4	0.954
	Rural	56(6.8)	96(11.6)	35(4.2)	1.70(0.31)	1.19,2.43	**0.004**	1.70(0.31)	1.19,2.43	**0.004**
**Oral health literacy**	Inadequate	187(22.7)	262(31.8)	76(9.2)	Ref.			Ref.		
	Marginal	59(7.1)	63(7.6)	5(0.6)	0.62(0.12)	0.42,0.92	**0.018**	0.62(0.13)	0.42,0.92	**0.018**
	Adequate	76(9.2)	90(10.9)	7(0.9)	0.81(0.17)	0.55,1.18	0.275	0.80(0.16)	0.55,1.18	0.275

^1^Ordered logistic regression

Coef.: Coefficient, CI: Confidence interval, SE: Standard error

The likelihood of good oral hygiene compared with moderate and fair oral hygiene was 40% higher in those with adequate OHL (P = 0.008). The likelihood of mild gingivitis compared with moderate and severe gingivitis was 20% higher in those with adequate OHL (P = 0.27).

## Discussion

This study was an OHL need assessment based on oral health behavior and socio-epidemiological phases of our planning model to design an educational intervention for enhancement of OHL. Technically, OHL interventions are necessarily required for our target population according to the results obtained from the OHL-AQ and clinical examination of participants. The results showed significant correlation of OHL and demographic factors with oral health behavior and oral health status in part. Smoking, frequency of dental visits, PI, and GI were correlated with OHL. However, these findings should be further confirmed in a randomized controlled clinical trial. The mean OHL score was generally low among the participants. Two-thirds of them had inadequate OHL, which was comparable to the results of other studies [6, 7, 25, 26]. The results also showed that promotion of OHL is an unmet need especially regarding making healthy decisions.

The participants in our study had previously received educational instructions as part of the Oral Health National Program in grades 1–6 of the primary school and had also received instructions regarding correct tooth brushing and cutting down the consumption of sugary snacks many times during their school years. Many of them were also part of the National Fluoride Therapy Program [27]. Thus, absence of a correlation between these two preventive behavior measures and OHL was unexpected. However, the PI (which can be considered as an outcome of toothbrushing) was significantly correlated with OHL; this finding has also been reported by some other studies [8, 9, 28]. Also, significant correlation of decayed teeth with OHL can be a delayed outcome of oral health behaviors.

Considering the current results regarding the presence of a significant association between OHL and clinical oral parameters, it may be suggested that an OHL intervention may be able to affect the mean number of decayed and missing teeth as well as the oral hygiene and gingival health status more than the mean number of filled teeth. Aside from the access to dental services, these findings may highlight the impact of socioeconomic factors as important barriers, which need to be addressed by promotion of OHL to pave the way towards the achievement of our goal, i.e. oral health promotion.

As shown in the present results, the demographic and socioeconomic factors are differently correlated with oral health behaviors and oral health status. It should be noted that the present study did not aim to scrutinize such relationships as they have already been confirmed in previous studies [4, 26, 29]. The main objective of this study was to focus on OHL as an important, yet less commonly addressed, part of oral health.

However, considering the relatively long course of caries development, the results may not be detectable by short-term assessment of dental indexes. But, a systematic review and a meta-analysis showed a significant correlation between dental caries and low level of oral hygiene; however, their results regarding the effects of OHL on gingival parameters, tooth extraction, and dental treatment needs were not clear [29, 30]. Although we found a significant correlation between GI and OHL, it should be noted that GI can be affected by systemic health and aging as well.

This study had some limitations. First, it should be emphasized that it was a cross-sectional study; thus, it cannot reveal the causal relationships. Despite the acceptable study power, our results are not generalizable to all communities at the national and international levels, and should be confirmed by further studies. The potential bias related to the questionnaire as recall bias was inevitable. There was also the possibility of reporting bias in smoking.

Our study had its strengths as well. The data were collected by using a self-reported questionnaire, and if there was an unanswered question, an interview would be performed. Thus, we did not have any missing data. OHL-AQ, as a valid and reliable tool for assessment of OHL was used for data collection in this study [17]. Targeting equal number of adult males and females was another strength of this study, which eliminated gender discriminations in this respect. Only limited studies on OHL have targeted equal number of adult males and females [8, 28]. Moreover, optimal intra-examiner reliability and clinical oral examination of all participants on a dental chair confirmed the method accuracy of the clinical data.

We also assessed the relationship of social status (educational, demographic, and economic aspects) and OHL with oral health behaviors. Moreover, we evaluated the epidemiology of oral health and diseases and their relationship with OHL in our selected target population. The authors will use the present results to design future interventions on OHL. Oral health can be enhanced by promotion of decision making, which is a neglected part of OHL. The study hypothesis (OHL intervention can improve oral health) will be examined in the next phase of the “Save Couples’ Smile” project. If this hypothesis is confirmed by a randomized controlled clinical trial, we may plan to integrate an OHL program in the PHC setting as a national health educational program for the newlyweds and young adults.

The present study as the first phase of the “Save Couples’ Smile” project was the first study conducted in Iran that assessed the oral health status as a final outcome in correlation with OHL according to dental and gingival parameters. To the best of the authors’ knowledge, “Save Couples’ Smile” is also the first OHL interventional project in Iran, and one of the few worldwide.

## Conclusion

We assessed our participants’ OHL and its association with their socio-demographic characteristics as determinants of oral health status and preventive behavior measures. Our results regarding the presence of a significant correlation between the OHL and oral health clinical outcomes can confirm the role of OHL with more emphasis on the decision making in oral health promotion. This finding further encouraged us to design an effective intervention for behavioral change in adults with further emphasis on behaviors related to OHL such as not smoking and inclusion of preventive dental services. The primary knowledge acquired through the social and epidemiological phases of the Precede-Proceed planning model can help us design beneficial educational programs at the individual level and design an intervention for oral health promotion at the community level. OHL is an unmet need of young couples, and related interventions could be necessary to promote their own oral health as well as the oral health of their future children. This intervention will be tested in a randomized control trial in the next phase of our project.

## Supporting information

S1 Checklist STROBE statement—Checklist of items that should be included in reports of *cross-sectional studies*. (DOC)

S1 Data (SAV)
