# Supplementary material for: Oral health literacy and socio-demographics as determinants of oral health status and preventive behavior measures in participants of a pre-marriage counseling program
Source: PLoS One. 2021 Nov 5;16(11):e0258810. doi: 10.1371/journal.pone.0258810 (PMC8570479; doi:10.1371/journal.pone.0258810)
Supplement: S1 Checklist — (DOC) [file pone.0258810.s001.doc]

STROBE Statement—Checklist of items that should be included in reports of ***cross-sectional studies***

|  | Item No | Recommendation |
| --- | --- | --- |
| **Title and abstract** | 1 | (*a*) Indicate the study’s design with a commonly used term in the title or the abstract  **It is indicated in the abstract line 1, page1.** |
|  |  | (*b*) Provide in the abstract an informative and balanced summary of what was done and what was found  **It is provided in the abstract, pages 1and 2.** |
| Introduction | | |
| Background/rationale | 2 | Explain the scientific background and rationale for the investigation being reported  **It is explained in paragraph 1 and 2 of the introduction, pages 2and 3.** |
| Objectives | 3 | State specific objectives, including any prespecified hypotheses  **It is specified in the last 3 lines of paragraph 4 of the introduction, page 4.** |
| Methods | | |
| Study design | 4 | Present key elements of study design early in the paper  **It is presented in the first line of materials and methods, page 4.** |
| Setting | 5 | Describe the setting, locations, and relevant dates, including periods of recruitment, exposure, follow-up, and data collection  **It is described in the first paragraph of materials and methods, page 4.** |
| Participants | 6 | (*a*) Give the eligibility criteria, and the sources and methods of selection of participants  **They are described in the second and third paragraphs of the materials and methods, page 5.** |
| Variables | 7 | Clearly define all outcomes, exposures, predictors, potential confounders, and effect modifiers. Give diagnostic criteria, if applicable  **They are defined in the paragraphs 5 and 6 of the materials and methods, pages 5 and 6.** |
| Data sources/ measurement | 8* | For each variable of interest, give sources of data and details of methods of assessment (measurement). Describe comparability of assessment methods if there is more than one group  **They are described in the paragraphs 5 and 6 of the materials and methods, pages 5 and 6**. |
| Bias | 9 | Describe any efforts to address potential sources of bias  **It is described in the last two lines page5.**  **It is also mentioned in the second paragraph of page 6.** |
| Study size | 10 | Explain how the study size was arrived at  **It is described in the first paragraph of page 5**. |
| Quantitative variables | 11 | Explain how quantitative variables were handled in the analyses. If applicable, describe which groupings were chosen and why  **They are explained in the paragraphs 5 and 6 of the materials and methods, pages 5 and 6**. |
| Statistical methods | 12 | (*a*) Describe all statistical methods, including those used to control for confounding  **It is explained in the last paragraph of the methods, page7**. |
|  |  | (*b*) Describe any methods used to examine subgroups and interactions  **It is explained in the last paragraph of the methods, page7.** |
|  |  | (*c*) Explain how missing data were addressed  **This is indirectly explained in the last two lines page5.** |
|  |  | (*d*) If applicable, describe analytical methods taking account of sampling strategy  **Not applicable.** |
|  |  | (*e*) Describe any sensitivity analyses  **Not applicable.** |
| Results | | |
| Participants | 13* | (a) Report numbers of individuals at each stage of study—eg numbers potentially eligible, examined for eligibility, confirmed eligible, included in the study, completing follow-up, and analysed  **They were reported in the first paragraph of results , page7.** |
|  |  | (b) Give reasons for non-participation at each stage  **Not applicable.** |
|  |  | (c) Consider use of a flow diagram  **Not applicable.** |
| Descriptive data | 14* | (a) Give characteristics of study participants (eg demographic, clinical, social) and information on exposures and potential confounders  **They are given in the first and second paragraphs of results, pages7 and 8 and also in the first columns of the tables.** |
|  |  | (b) Indicate number of participants with missing data for each variable of interest  **Not applicable.** |
| Outcome data | 15* | Report numbers of outcome events or summary measures  **They are reported in tables.** |
| Main results | 16 | (*a*) Give unadjusted estimates and, if applicable, confounder-adjusted estimates and their precision (eg, 95% confidence interval). Make clear which confounders were adjusted for and why they were included  **They are reported in tables.** |
|  |  | (*b*) Report category boundaries when continuous variables were categorized  **They are explained in the methods.** |
|  |  | (*c*) If relevant, consider translating estimates of relative risk into absolute risk for a meaningful time period  **Not applicable.** |
| Other analyses | 17 | Report other analyses done—eg analyses of subgroups and interactions, and sensitivity analyses  **Interactions are noticed.** |
| Discussion | | |
| Key results | 18 | Summarise key results with reference to study objectives  **They are summarised in the first paragraph of discussion, page 19.** |
| Limitations | 19 | Discuss limitations of the study, taking into account sources of potential bias or imprecision. Discuss both direction and magnitude of any potential bias  **They are discussed in the last paragraph ,page 20 and in the first paragraph ,page 21.** |
| Interpretation | 20 | Give a cautious overall interpretation of results considering objectives, limitations, multiplicity of analyses, results from similar studies, and other relevant evidence  **They are discussed in discussion paragraphs, pages 19-21.** |
| Generalisability | 21 | Discuss the generalisability (external validity) of the study results  **They are discussed in the last paragraph ,page 20.** |
| Other information | | |
| Funding | 22 | Give the source of funding and the role of the funders for the present study and, if applicable, for the original study on which the present article is based  It is mostly funded by the authors. |

*Give information separately for exposed and unexposed groups.

**Note:** An Explanation and Elaboration article discusses each checklist item and gives methodological background and published examples of transparent reporting. The STROBE checklist is best used in conjunction with this article (freely available on the Web sites of PLoS Medicine at http://www.plosmedicine.org/, Annals of Internal Medicine at http://www.annals.org/, and Epidemiology at http://www.epidem.com/). Information on the STROBE Initiative is available at www.strobe-statement.org.
